# How I do it: Optic nerve decompression in patient with osteopetrosis

**DOI:** 10.1007/s00701-025-06513-8

**Published:** 2025-04-09

**Authors:** Katarína Horčičáková, Jana Táborská, Adéla Bubeníková, Vladimír Beneš

**Affiliations:** Department of Neurosurgery, Second Faculty of Medicine, Charles University and Motol University Hospital, Prague, Czech Republic

**Keywords:** Osteopetrosis, Optic nerve, Decompression, Anterior clinoidectomy

## Abstract

**Background:**

Osteopetrosis is a rare genetic disorder causing excessive skeletal density, with a predilection to affect the skull base. This commonly leads to optic canal stenosis, optic nerve compression, and atrophy and vision loss. Timely optic nerve decompression can be an effective surgical intervention to preserve vision.

**Methods:**

Pterional approach combining both extra- and intradural optic nerve decompression was performed. Intradural optic nerve identification proved a helpful and safe way to facilitate adequate decompression while dissecting in the region of hypertrophic anterior clinoid process.

**Conclusion:**

Precise surgical technique and anatomical awareness are crucial for minimizing complications and aiding recovery.

**Supplementary Information:**

The online version contains supplementary material available at 10.1007/s00701-025-06513-8.

## Case summary

A five-year old girl with a genetically proven Osteopetrosis type II presented with bilateral vision worsening. The left eye was affected more and was addressed first, the right eye 6 weeks later. Preoperative visual exam included visual acuity (decreased bilaterally left > right), fundoscopy (pale disc), visual evoked potentials (increased N70 latency) and optical coherence tomography (Retinal Nerve Fiber Layer – left 31 µm, right 38 µm); visual fields were not performed due to poor cooperation.

## Relevant surgical anatomy

The optic nerve can be divided into four segments: intraocular, intraorbital, intracanalicular and intracranial. The intracanalicular segment, approximately 10 mm long, traverses the optic canal (OC) alongside the ophthalmic artery. After exiting the OC, it passes through the suprasellar cistern to merge with the contralateral optic nerve, forming the optic chiasm. The OC connects the middle cranial fossa to the orbital apex and is enclosed by the falciform ligament of the dura mater. The roof comprises the lesser wing of the sphenoid bone, while the optic strut forms the lateral wall, separating the OC from the superior orbital fissure (SOF). The optic strut, lesser wing of the sphenoid, and the OC roof anchor the anterior clinoid process (ACP). Accessing the ACP necessitates opening the SOF and dissecting the lateral wall of the cavernous sinus. The meningo-orbital band, a dural fold tethering the fronto-temporal dura to the periorbita located in the most lateral part of the SOF, must be transected to fully expose the SOF.

## Positioning and skin incision

**Fig. 1:**
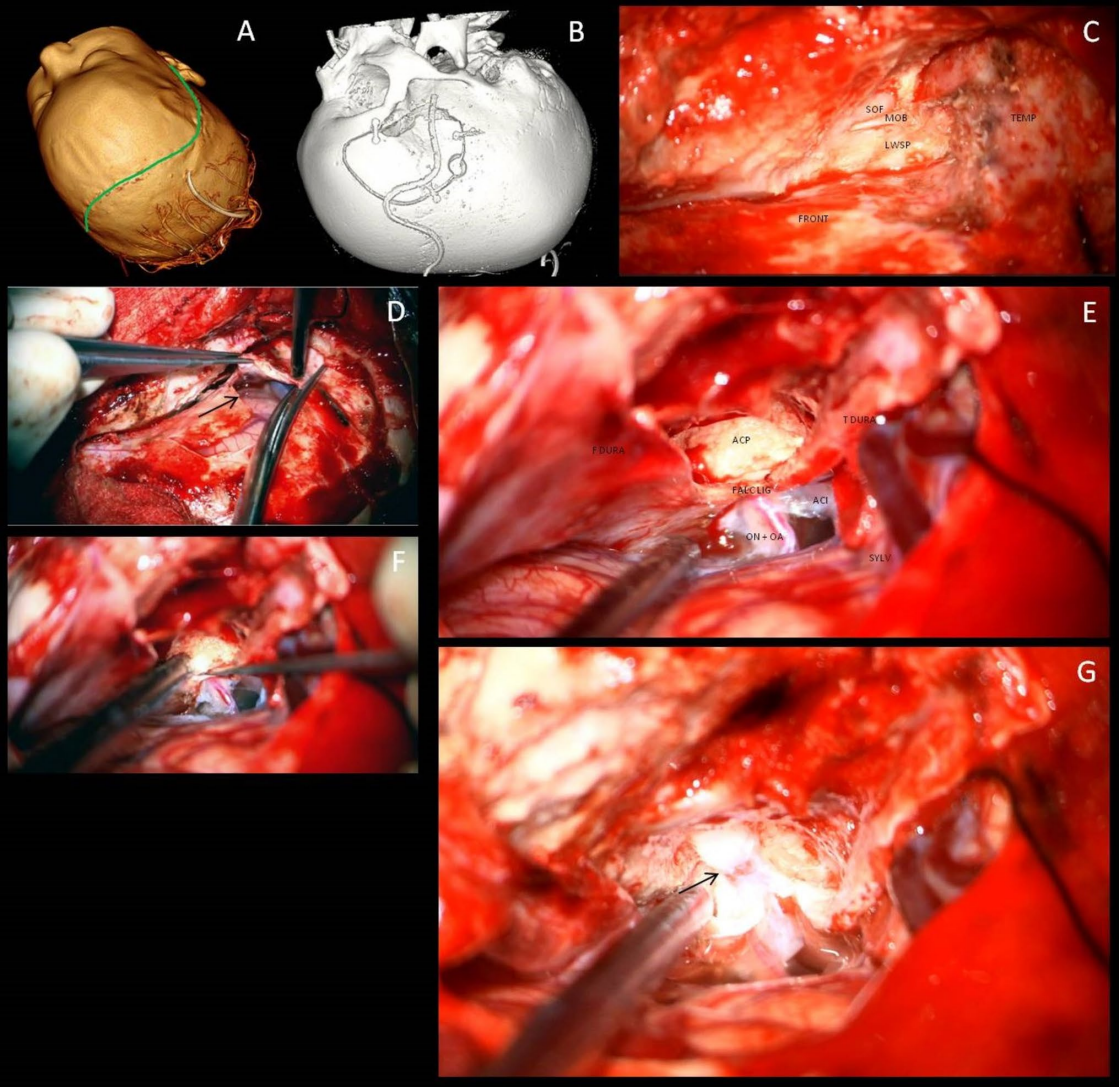
**A** The patient is positioned supine with the head slightly extended and rotated 45° to the contralateral side. Skin incision is marked by a green line. **B**—Extent of craniotomy. **C**—Following pterional craniotomy, the following structures are identified: frontal lobe (FRONT), temporal lobe (TEMP), superior orbital fissure (SOF), meningo-orbital band (MOB) and lesser wing of the spenoid bone (LWSP). **D**—A vertical durotomy parallel to the Sylvian fissure is performed with a its limb (arrow) extending towards the region of the anterior clinoid process (ACP). **E**—Intradural dissection identifies the optic nerve (ON) and ophthalmic artery (OA), internal carotid artery (ACI), Sylvian fissure (SYLV), falciform ligament (FALC LIG) and ACP. **F**—The roof of the optic canal is identified using a fine dissector. **G**—Following ACP removal and medial ON decompression the site of severe compression is visible (arrow)

Following administration of anesthesia, a lumbar drain was inserted to facilitate brain relaxation. The patient was positioned supine, with the head slightly elevated, rotated 45° contralaterally, and secured in a Mayfield clamp (Fig. 1A). Intraoperative navigation guided anatomical identification and optimized the surgical trajectory. A skin incision was made 1 cm anterior to the tragus, anterior to the superficial temporal artery and auriculotemporal nerve, curving upward slightly behind the hairline, and extending beyond the midline. The incision was to be later extended contralaterally in the same fashion during contralateral procedure.

## Craniotomy

A standard pterional craniotomy was performed (Fig. 1B). Two burr holes were placed in the keyhole region and in the squamous part of the temporal bone. These were connected using a craniotome along a curved trajectory. Care was taken to provide flush access to the frontal cranial base.

## Description of the technique

Following the craniotomy, the lesser wing of the sphenoid was drilled to expose and transect the meningo-orbital band (Fig. 1C). The SOF was decompressed and the lateral wall of the cavernous sinus was peeled away to access the ACP. The bone was notably hypertrophic. The lesser wing of the sphenoid bone was drilled away. Despite the frequent use of neuronavigation and due to the excessive bone mass, a complete extradural clinoidectomy was deemed unsafe as extradural optic nerve identification proved challenging.

To enhance visual control and facilitate early optic nerve identification, a vertical durotomy parallel to the Sylvian fissure extending to the region of the ACP was performed (Fig. 1D, E). Subfrontal dissection revealed the optic nerve, internal carotid artery (ICA) and ophthalmic artery. The durotomy was then extended toward the falciform ligament, linking the extra- and intradural space in the ACP region.

The OC roof was identified (Fig. 1F) and drilled using a fine diamond drill under continuous irrigation. Afterwards, the optic strut was also drilled and the remaining ACP was removed. Medial to the optic nerve, the adjacent bone of the planum sphenoidale was drilled away as well, to achieve a 180° decompression. The falciform ligament was transected. A 7 mm segment of the optic nerve appeared hyperemic, indicating compression within the stenotic canal (Fig. 1G). The dura was closed and the craniotomy flap was replaced. To prevent thermal injury, the field was continuously irrigated during drilling. Following durotomy, papaverine solution was applied regularly to improve optic nerve perfusion.

**Fig. 2:**
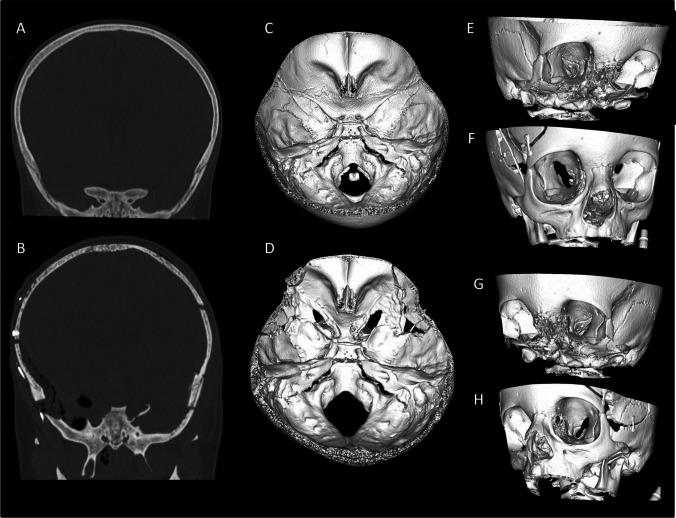
Preoperative (**A**, **C**, **E**, **G**) and postoperative (**B**, **D**, **F**, **H**) computer tomography. Coronal section shows complete clinoidectomy on the right side and nearly complete clinoidectomy on the left side (**B**). The extent of preoperative stenosis of the optic canal visible on 3D reconstruction of the skull base (**C**), viewed from the right (**E**) and left (**G**) orbit. The extent of decompression from similar views can be appreciated (**D**, **F**, **H**)

Postoperatively (Fig. 2), visual function remained stable in both eyes (Retinal Nerve Fiber Layer – left 58 µm, right unchanged), confirming the procedure’s safety and effectiveness.

## Indications

The optimal timing for OC decompression has not yet been clearly established, with most procedures performed after significant vision loss. However, some reports suggests greater efficacy in older patients with milder osteopetrosis [3], and highlights the importance of early intervention in autosomal dominant cases to prevent vision loss [4]. We staged the surgery into two procedures starting with the more affected eye. If the procedure led to unfortunate worsening of vision, the child would still have function of the better eye (and additional surgery would probably be contraindicated after thorough discussion with the family). There is no recommendation as to the timing of the contralateral decompression, we waited arbitrary 6 weeks for sufficient recovery. Bilateral simultaneous decompression has also been described [1].

## Limitations

The exact mechanism of optic nerve injury in patients with osteopetrosis remains unclear. It is primarily attributed to mechanical compression within the narrowed OC [6]. Alternative theories include primary optic nerve dysmyelination, atrophy due to papilledema from retinal vein compression, primary elevated intracranial pressure, and hydrocephalus [7, 8]. Adding to this complexity, Hoyt and Billson reported three patients who had diffuse retinal degeneration shown by an electroretinography scan and after the decompression, one of their three patients continued to have a progressive loss of eyesight [2].

In contrast, Al-Mefty et al. found visual improvement in five of six patients following bilateral transfrontal optic nerve decompression [1].

Given the possibly complex etiology of visual decline in osteopetrosis patients, even after adequate decompression, progression of visual loss may occur. Another limitation is the worse cooperation during certain ophthalmological examinations encountered in very young children.

## How to avoid complications

Optic canal decompression requires drilling hypertrophic bone while preventing thermal injury and preserving neurovascular structures. Essential precautions include continuous saline irrigation, intermittent drilling, and diamond burr use.

To mitigate ischemic optic nerve damage, local papaverine application is recommended for its vasodilatory properties.

Corticosteroids aid in reducing edema and preventing postoperative inflammation and ischemia. Due to the proximity of the oculomotor, trochlear, abducens, and ophthalmic trigeminal nerves, meticulous dissection around the superior orbital fissure is crucial.

## Specific information for the patient

Extensive microsurgical optic nerve decompression is a safe and effective method for preserving or enhancing vision in patients with osteopetrosis. However, surgery near the cavernous sinus or orbital apex may inadvertently affect surrounding cranial nerves, resulting in facial muscle weakness, ptosis, diplopia or visual function worsening. It is important to inform the patients and parents about these potential risks prior to the procedure.

## key points summary

- Optic nerve decompression is a safe and effective procedure for maintaining or improving eyesight in patients with osteopetrosis. Pre- and postoperative visual exam should include at least visual acuity, fundoscopy, visual evoked potentials, optical coherence tomography and visual fields.
- Use of lumbar drain facilitates extradural access to the ACP region.
- Pterional craniotomy provides safe and sufficient access to the optic canal.
- Vertical durotomy parallel to the Sylvian fissure towards the ACP region, combined with intradural clinoidectomy, provides early visual control and identification of the optic nerve.
- Maximal optic nerve decompression requires drilling of the bone along the entire length of the optic nerve.
- The use of diamond burrs, frequent pauses during drilling, and continuous irrigation are essential for minimizing thermal injury to surrounding neurovascular structures.
- We recommend the local use of papaverine to improve local perfusion, as well as the administration of corticosteroids to minimize edema.
- Caution is essential when exposing the SOF due to the close proximity of its neural content.
- Potential risks that may arise include worsening of visual function, facial muscle weakness, ptosis or diplopia, resulting from cranial nerve injury.
- Patients should be well-informed about the procedure’s benefits, potential risks, and expected outcomes before surgery.

## Supplementary Information

Below is the link to the electronic supplementary material.

Supplementary file1 (MP4 166303 KB)

## Data Availability

No datasets were generated or analysed during the current study.
